# The Association between Two MicroRNA Variants (miR-499, miR-149) and Gastrointestinal Cancer Risk: A Meta-Analysis

**DOI:** 10.1371/journal.pone.0081967

**Published:** 2013-11-29

**Authors:** Li Li, Yunjian Sheng, Lin Lv, Jian Gao

**Affiliations:** 1 Department of Gastroenterology, The Second Affiliated Hospital, Chongqing Medical University, Chongqing, China; 2 Department of Infectious Diseases, The Affiliated Hospital of Luzhou Medical College, Luzhou, China; Duke-NUS, Singapore

## Abstract

**Background:**

MicroRNAs (miRNAs) are small RNA molecules that regulate the expression of corresponding messenger RNAs (mRNAs). Single nucleotide polymorphisms (SNPs) in miRNAs may contribute to cancer susceptibility due to changes in the microRNA’s properties and/or maturation. The present study aimed to investigate the association between two miRNA polymorphisms (miR-499 rs3746444 and miR-149 rs2292832) and gastrointestinal (GI) cancer risk.

**Methodology/Principal Findings:**

We conducted a search of case-control studies in PubMed, Wiley Online Library, Web of Science and the CNKI database. Eleven rs3746444 studies and six rs2292832 studies were included in our meta-analysis. The only obvious association between the miR-499 polymorphism and colorectal cancer susceptibility was found in the homozygote comparison (GG vs. AA: OR = 1.66, 95% CI: 1.02–2.70, *P*_h_ = 0.10, *P* = 0.04). No signiﬁcant association was found in the subgroup analysis for ethnicity and risk of hepatocellular and gastric cancer. A marginally elevated GI cancer risk was discovered in the recessive model for miR-149 (TT vs. TC+CC: OR = 1.15, 95% CI: 1.03–1.30, *P*_h_ = 0.68, *P* = 0.02). Stratifying the results by ethnicity revealed a slight association between the recessive model and the Asian population (TT vs. TC+CC: OR = 1.14, 95% CI: 1.01–1.29, *P*_h_ = 0.79, *P* = 0.03).

**Conclusions/Significance:**

The present meta-analysis indicates that miR-499 may be associated with the risk to colorectal cancer. MiR-149 may confer a marginally increased risk of susceptibility to gastrointestinal cancer, especially for Asians.

## Introduction

MicroRNAs (miRNAs) are a subset of short, endogenous non-coding RNAs that play key roles in controlling the expression of many cellular proteins. It has been estimated that a single miRNA can potentially target hundreds of mRNAs and that almost 30% of the protein-coding genes in the human genome can be regulated by miRNAs [1]. To date, more than 1200 miRNA sequences have been identified in humans; however, specific functions have not yet been identified for most of these sequences. MiRNAs can function in the deregulation of important genes that play key roles in tumorigenesis, tumor development, and angiogenesis or can have oncogenic or tumor suppressor roles [2]. MiRNAs that are located in chromosomal regions that are amplified in cancers can function as oncogenes, while miRNAs located in regions that are deleted in cancers may act as tumor suppressors [3,4]. Evidence has also shown that a global reduction in miRNA processing increases cancer susceptibility, and miRNA profiling has been successfully used to classify tumors [4,5].

Single-nucleotide polymorphisms (SNPs) or mutations in miRNA sequences may alter miRNA expression and/or maturation, in addition to changing the effects of miRNAs on their target genes. SNPs are the most common type of genetic variation and are associated with population diversity, disease susceptibility and individual response to medicine [6]. SNPs may disrupt miRNA-target interaction, resulting in the deregulation of target gene expression, as shown in non-small cell lung cancer [7]. Hoffman et al. revealed that miR-196a-2 might have a potentially oncogenic role in breast tumorigenesis, and a functional genetic variant in the mature region of miR-196a-2 could serve as a novel biomarker for breast cancer susceptibility [8]. In other study, Zeng et al. found that the rs2910164 SNP in miR-146a was associated with an elevated risk of gastric cancer in the Chinese population [9]. In contrast, none of the 40 miRNA-related gene polymorphisms were identified as independent prognostic markers for Korean patients with surgically resected colorectal cancer [10].

 The role of genetic variants in miRNAs on GI cancer susceptibility remains largely unknown. Several recent reports identified an association between two genetic miRNA variants (miR-499 rs3746444 and miR-149 rs2292832) and the risk of gastrointestinal cancer. For example, Xiang et al. found that individuals with the miRNA-499 GG genotype were about threefold more susceptible to hepatocellular carcinoma (HCC) (OR = 3.63, 95% CI: 1.545–8.532) than individuals with the AA genotype [11]. In contrast, Kim et al. demonstrated that individuals with the AG+GG genotypes of the miR-499 A>G rs3746444 variant have a signiﬁcantly lower risk of HCC than individuals with the AA genotype. Patients with the miR-149C>T CT and CT+CC genotypes have a significantly reduced risk of HCC (CT; AOR = 0.542, 95% CI: 0.332–0.886, CT+CC; AOR = 0.536, 95% CI: 0.335–0.858) [12]. A study in Taiwan found no significant association between miRNA149 gene polymorphisms and the risk of oral cancer [13]. These results are inconsistent and unreliable. Therefore, we performed a meta-analysis of all of the eligible studies to obtain a more precise assessment of the association between these two SNPs and the risk of gastrointestinal cancers.

## Materials and Methods

### Publication search

We searched PubMed, Wiley Online Library, Web of Science and the CNKI database for studies published between January 1, 2000 and January 1, 2013. The search was limited to humans. The keywords used in the search included: "microRNA or miRNA", "cancer or tumor, gene or polymorphism or variation" and "miR-499 or rs3746444，miR-149 or rs2292832". Only published studies with full text articles were included. All of the studies matching the eligibility criteria were included in our meta-analysis. 

### Inclusion and exclusion criteria

The studies included in the meta-analysis met the following criteria: 1) The study was designed as a case-control study, 2) The association between the miR-499 polymorphism, miR-149 polymorphism and gastrointestinal cancer risk was explored and 3) The study contained sufficient data for the computation of odds ratios and corresponding 95% confidence intervals (ORs, 95% CIs). Non-original articles, non-case–control studies, studies that duplicated previous publications, studies involving cancer cells and studies investigating animal models were excluded. 

### Data extraction

We extracted the necessary data from the ﬁnal eligible articles independently using the inclusion criteria listed above. The following information was extracted from each of the included articles: the name of first author, the year of publication, the country of origin, patient ethnicity (Caucasian, Asian or other), cancer type, the genotyping method, the total number of cases and controls, the number of genotyped cases and controls and *P*-values for Hardy-Weinberg equilibrium (HWE) of control groups.

### Quality assessment

The quality of the studies was independently assessed using a set of predetermined criteria that was extracted and modified from previous studies [14,15,38](Table 1). These scores were based on traditional epidemiological considerations and cancer genetics issues. The scores ranged from a low of zero to a high of 18 with higher scores presented better quality. Those articles scoring < 12 were classified as ‘‘low quality’’, and those articles scoring ≥12 were considered ‘‘high quality’’.

**Table 1:** Scale for quality assessment.

**Criterion**	**Score**
Source of cases	
Selected from population or cancer registry	3
Selected from hospital	2
Selected from pathology archives, but without description	1
Not described	0
Source of controls	
Population-based	3
Blood donors or volunteers	2
Hospital-based (cancer-free patients)	1
Not described	0
Case-control match	
Matched by age and gender	3
Not matched by age and gender	0
Specimens used for determining genotypes	
White blood cells or normal tissues	3
Tumor tissues or exfoliated cells of tissue	0
Hardy-Weinberg equilibrium in controls	
Hardy-Weinberg equilibrium	3
Hardy-Weinberg disequilibrium	0
Total sample size	
>1000	3
>500 and <1000	2
>200 and <500	1
<200	0

### Statistical methods

We used Cochrane Review Manager Version 5.1 (http://ims.cochrane.org/revman/download) and Stata/SE software 12.0 (Stata Corporation, College Station, Texas) to analyze the data from each study. ORs and 95% CIs were calculated to calculate the strength of the association between the two SNPs (miR-499 rs3746444，miR-149 rs2292832) and the susceptibility to GI cancer using the reported allele and genotype frequencies in the cases and controls. The pooled ORs were calculated for the genetic models (G versus A), homozygote comparison (GG versus AA), heterozygote comparison (AG versus AA), dominant model (GG+AG versus AA), and recessive model (GG versus AG+AA), as was miR-149. Subgroup analyses were performed for racial descent and tumor type.

 The significance of the pooled ORs was determined using the Z-test; a P value<0.05 was considered statistically significant. A chi-square-based Q-test was performed to check for heterogeneity. A P value greater than 0.10 in the Q-test indicates no significant heterogeneity among studies, thereby permitting a fixed-effects model to be used to calculate the combined OR. If the P value of the Q tests is below 0.10, a random-effects model could be more suitable. The *I* ^2^ index expresses the percentage of the total cross-study variation that occurs due to heterogeneity. *I* ^2^ values of 25, 50 and 75% were used as evidence of low, moderate, and high heterogeneity, respectively. Hardy-Weinberg equilibrium (HWE) was evaluated for each study by using the Chi-square test in the control groups. A P value<0.05 was considered indicative of a departure from HWE. Furthermore, publication bias was assessed using Egger’ s linear regression method and Begg’s funnel plot (statistical significance was deﬁned as *P*<0.05).

## Results

### Characteristics of the studies

**Figure 1:**
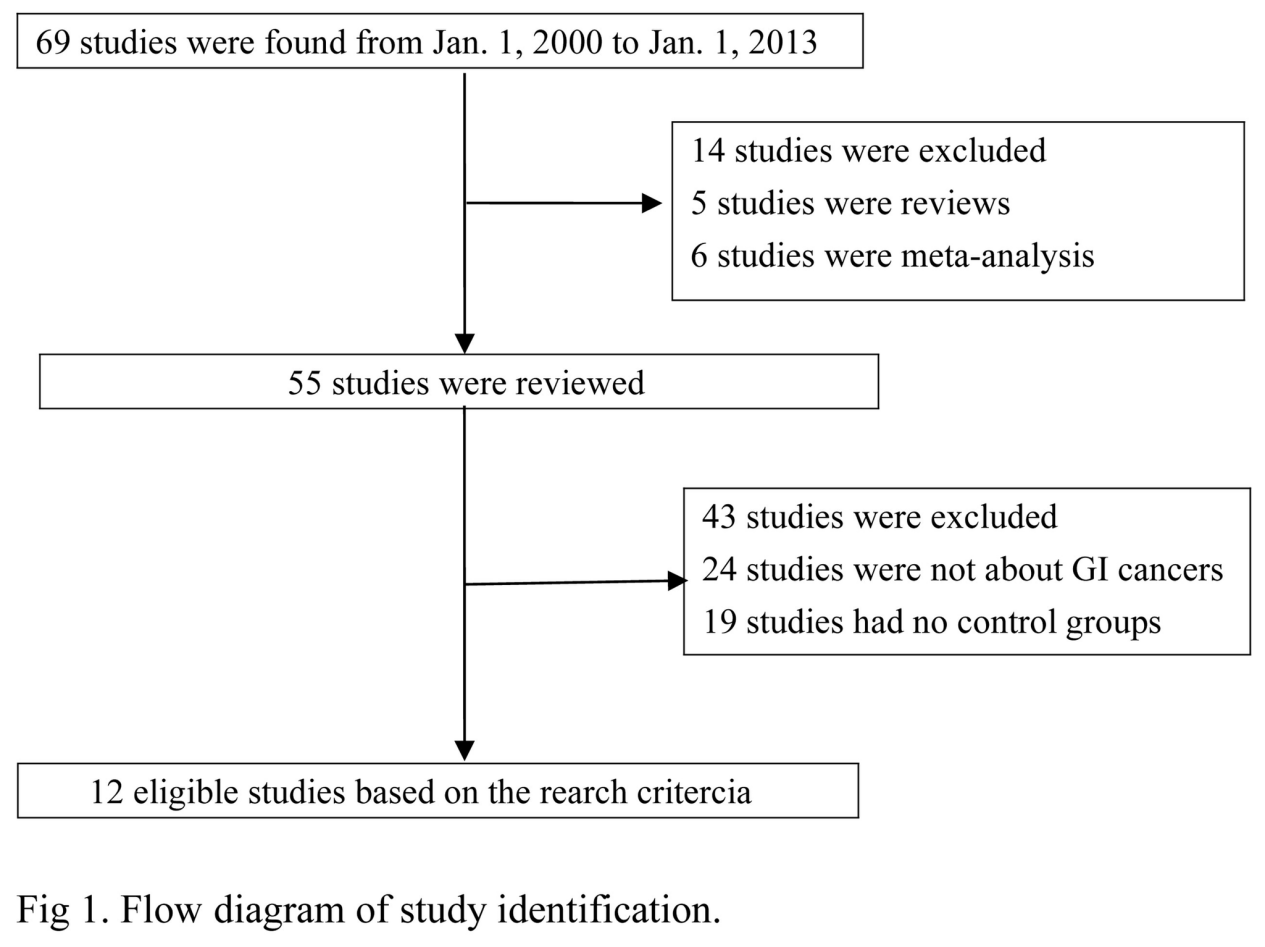
Flow diagram of study identification.

Of the 69 studies initially identified, 5 studies were reviews, 9 studies were meta-analyses, 24 studies were not about GI cancers, and 19 studies did not have a control group. Therefore, 12 case-control studies were included in this meta-analysis. The flow chart in Figure 1 summarizes this literature review process. A total of 11 studies [11–13,16–23] involving 3,275 cases and 3,794 controls were ultimately analyzed for miR-499, and 6 studies [12,13,21–24] involving 2,413 cases and 2,457 controls were analyzed for miR-149. Two of the miR-499 studies investigated Caucasians and nine studies investigated Asians. Only one miR-149 study investigated Caucasians, while 5 studies investigated Asians. We considered patients with oral cancer as separate group and pooled these patients into the quantitative analysis independently. All of the controls in the studies were cancer free and matched for sex and age. The characteristics of the selected studies are summarized in Table 2 and the genotype frequency distribution was shown in Table S1. The Hardy-Weinberg Equilibrium test of control groups was shown in Table S2 .

**Table 2:** Characteristics of 12 case-control studies on miR-499 and miR-149.

**Author**	**Year**	**Ethnicity**	**Cancer type**	**Control source**	**Genotyping method**	**SNP**	**Case /Control**	**HWE of control**	**Quality** **score**
Xiang	2012	Asian	HCC	HB	PCR-RFLP	rs3746444	100/100	0.28	10
Zhou	2012	Asian	HCC	HB	PCR-RFLP	rs3746444	186/483	0.10	14
Kim	2012	Asian	HCC	PB	PCR-RFLP	rs3746444	159/201	0.27	13
				PB	PCR-RFLP	rs2292832	159/201	0.34	13
Akkiz	2011	Caucasian	HCC	HB	PCR-RFLP	rs3746444	222/222	0.03	13
Ahn	2012	Asian	GC	PB	PCR-RFLP	rs3746444	461/447	0.82	13
				PB	PCR-RFLP	rs2292832	461/447	0.97	13
Okubo	2010	Asian	GC	HB	PCR-RFLP	rs3746444	552/697	0.04	9
Vinci	2012	Caucasian	CRC	NR	HRMA	rs3746444	160/178	0.02	10
				NR	HRMA	rs2292832	160/178	0.91	13
Min	2011	Asian	CRC	PB	PCR-RFLP	rs3746444	446/502	0.45	14
				PB	PCR-RFLP	rs2292832	446/502	0.95	14
Srivastava	2010	Asian	GBC	PB	PCR-RFLP	rs3746444	230/230	0.56	15
Chu	2012	Asian	OSCC	HB	PCR-RFLP	rs3746444	470/425	0.97	11
				HB	PCR-RFLP	rs2292832	470/425	<0.01	8
Umar	2012	Asian	ESCC	PB	PCR-RFLP	rs3746444	289/309	0.08	13
Zhang	2012	Asian	CRC	NR	PCR-RFLP	rs2292832	435/443	0.58	13
			GC	NR	PCR-RFLP	rs2292832	274/269	0.69	13

CRC: colorectal cancer; ESCC: esophageal squamous cell carcinoma; GBC: gallbladder cancer; GC: gastric cancer; HCC: hepatocellular cancer; OSCC: oral cavity squamous cancer; HWE: Hardy-Weinberg equilibrium in control groups; PCR-RFLP: polymerase chain reaction-restriction fragment length polymorphism; HRMA: high-resolution melting analysis; HB: hospital based; PB: population based; NR: not reported.

### Meta-analysis results

There was no evidence of an association between the miR-499 A>G polymorphism and GI cancers in the four genetic models and the allele contrast when all types of cancer were considered together in the meta-analysis (all *P*-values >  0.05, Table 3). However, as shown in Table 3, the risk of colorectal cancer was increased in the homozygote comparison when the analysis was stratified by tumor type (GG versus AA: OR = 1.66, 95% CI: 1.02–2.70, *P*_h_ = 0.10, *P* = 0.04)(Table 3). We were unable to identify a significant association between the miR-499 rs3746444 polymorphism and hepatocellular cancer (G versus A: OR = 1.12, 95% CI: 0.77–1.62, *P*_h_ = 0.004, *P*= 0.55; GG+AG versus AA: OR = 1.09, 95% CI: 0.73–1.64, *P*_h_ = 0.02, *P* = 0.67; GG versus AG+AA: OR = 1.26, 95% CI: 0.92–1.73, *P*_h_ = 0.11, *P* = 0.14; GG versus AA: OR = 1.27, 95% CI: 0.60- 2.69, *P*_h_ = 0.04, *P* = 0.54; AG versus AA: OR = 0.99, 95% CI: 0.78–1.26, *P*_h_ = 0.16, *P* = 0.96) (Table 3). Similarly, the effect was also non-significant in the gastric cancer group. Ethnicity was also taken into consideration in the subgroup analysis. There was no significant association between the miR-499 rs3746444 polymorphism and GI cancer risk for the four genetic models and the allele contrast in Asians [(G versus A: OR = 1.11, 95% CI: 0.91–1.35, *P*_h_ < 0.0001, *P* = 0.30), (GG+AG versus AA: OR = 1.09, 95% CI: 0.88–1.35, *P*_h_ = 0.0003, *P* = 0.41), (GG versus AG+AA: OR = 1.27, 95% CI: 0.99–1.63, *P*_h_ = 0.10, *P* = 0.06), (GG versus AA: OR = 1.26, 95% CI: 0.84–1.89, *P*_h_ = 0.03, *P* = 0.27), (AG versus AA: OR = 1.06, 95% CI: 0.87–1.28, *P*_h_ = 0.006, *P* = 0.57)]. A similar result was also observed in Caucasians (G versus A: OR = 1.20, 95% CI: 0.97–1.48, *P*_h_ = 0.29, *P* =0.09)(Table 3). 

**Table 3:** Meta-analysis of miR-499 and miR-149 with gastrointestinal cancer susceptibility.

**MiR-499**		**GG vs AA**		**AG vs AA**		**G vs A**		**GG+AG vs AA**		**GGvs AG+AA**	
	**N**	**OR (95%CI**)	***P*_h_**	**OR (95%CI**)	***P*_h_**	**OR (95%CI)**	***P_h_***	**OR (95%CI**)	***P*_h_**	**OR (95%CI**)	***P*_h_**
**Total**	11	1.33(0.96,1.85)	0.03	1.02(0.85,1.21)	0.006	1.13(0.96,1.33)	<0.0001	1.08(0.90,1.30)	0.001	1.34(0.99,1.80)	0.04
**Cancer type**											
**HCC**	4	1.27(0.60,2.69)	0.04	0.99(0.78,1.26)	0.16	1.12(0.77,1.62)	0.004	1.09(0.73,1.64)	0.02	1.26(0.92,1.73)	0.11
**GC**	2	1.28(0.85,1.93)	0.42	0.92(0.76,1.11)	0.48	1.01(0.87,1.18)	0.22	0.96(0.80,1.15)	0.31	1.31(0.87,1.96)	0.47
**CRC**	2	**1.66(1.02,2.70)**	0.12	0.94(0.74,1.20)	0.10	1.14(0.94,1.37)	0.16	1.05(0.83,1.31)	0.96	1.64(0.61,4.43)	0.05
**Others**	3	1.39(0.51,3.78)	0.02	1.21(0.74,1.97)	0.007	1.25(0.75,2.00)	0.0001	1.23(0.72,2.11)	0.0005	1.33(0.55,3.21)	0.04
**Ethnicity**											
**Caucasian**	2	1.59(0.79,3.16)	0.09	0.80(0.56,1.14)	0.26	1.20(0.97,1.48)	0.29	1.05(0.76,1.43)	0.95	1.69(0.76,3.78)	0.03
**Asian**	9	1.26(0.84,1.89)	0.03	1.06(0.87,1.28)	0.006	1.11(0.91,1.35)	<0.0001	1.09(0.88,1.35)	0.0003	1.27(0.99,1.63)	0.10
**MiR-149**		**TT vs CC**		**TC vs CC**		**T vs C**		**TT+TC vs CC**		**TT vs TC+CC**	
	**N**	**OR (95%CI)**	***P*_h_**	**OR (95%CI)**	***P*_h_**	**OR (95%CI)**	***P*_h_**	**OR (95%CI)**	***P*_h_**	**OR (95%CI)**	***P*_h_**
**Total**	7	1.02(0.84,1.24)	0.79	0.83(0.69,1.00)	0.99	1.06(0.97,1.16)	0.81	0.92(0.77,1.10)	0.98	**1.15(1.03,1.30)**	0.68
**Cancer type**											
**CRC**	3	1.07(0.81,1.42)	0.62	0.86(0.66,1.11)	1.00	1.06(0.94,1.21)	0.98	0.94(0.74,1.20)	0.99	1.16(0.97,1.38)	0.63
**GC**	2	0.99(0.70,1.40)	0.98	0.79(0.56,1.12)	0.63	1.07(0.92,1.25)	0.88	0.89(0.64,1.24)	0.79	1.18(0.96,1.45)	0.61
**Others**	2	0.95(0.62,1.46)	0.17	0.82(0.52,1.30)	0.54	1.07(0.76,1.51)	0.09	0.90(0.59,1.36)	0.31	1.11(0.87,1.42)	0.10
**Ethnicity**											
**Asian**	6	0.99(0.81,1.21)	0.85	0.83(0.67,1.02)	0.98	1.06(0.97,1.16)	0.71	0.91(0.75,1.11)	0.95	**1.14(1.01,1.29)**	0.69

*P*_h_: *P* value of Q-test for heterogeneity test.Random-effects model was used when a P value<0.10 for heterogeneity test; otherwise, fixed-effects model was used.

**Figure 2:**
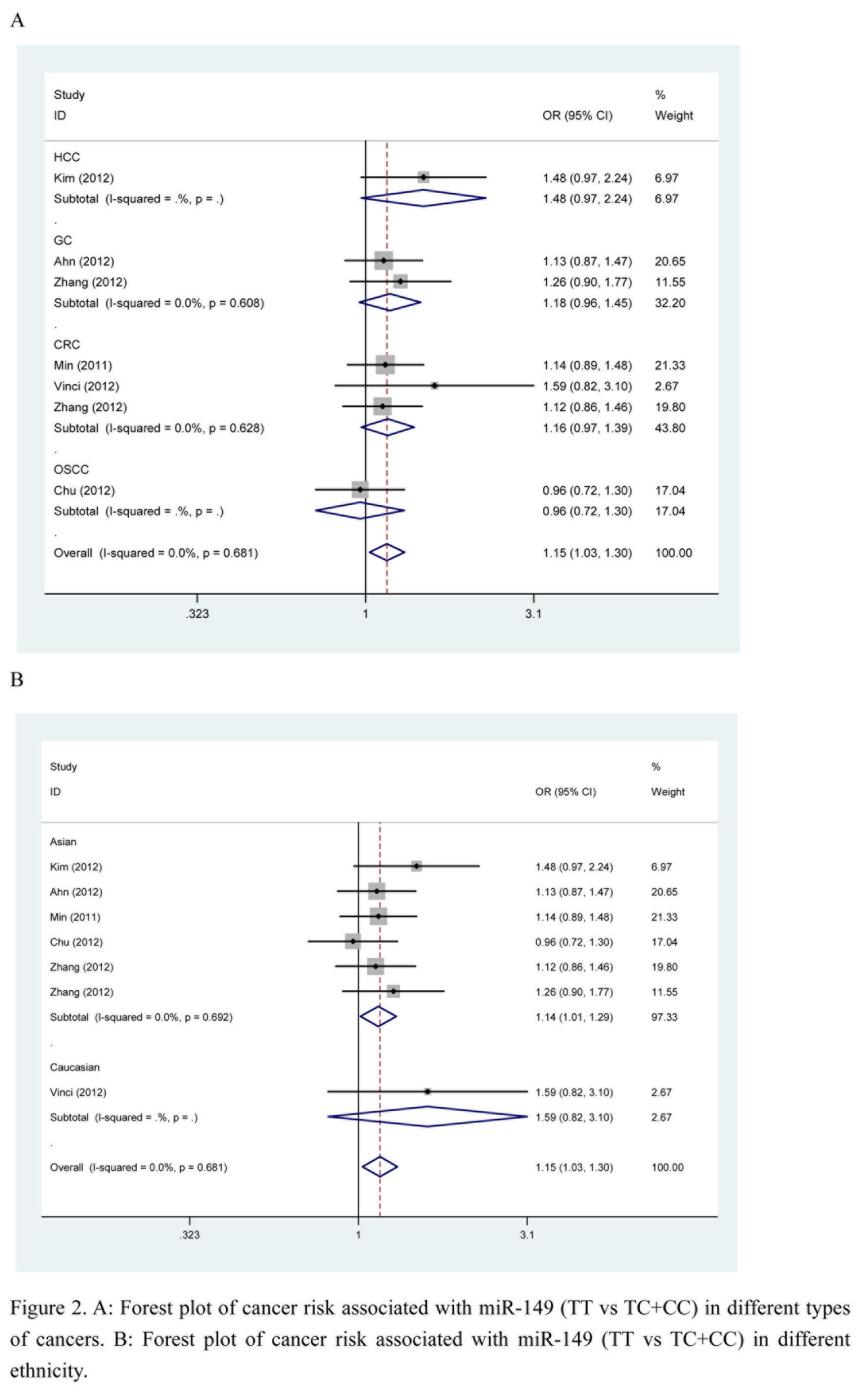
A: Forest plot of cancer risk associated with miR-149 (TT vs TC+CC) in different types of cancers. **B**: **Forest plot of cancer risk associated with miR-149 (TT vs TC+CC) in different ethnicity**.

For miR-149, there was a marginally increased overall risk of cancer in the recessive model (TT versus TC+CC: OR = 1.15, 95% CI: 1.03–1.30, *P*_h_ = 0.68, *P* = 0.02). A slight association was also found in Asian populations in the recessive model (TT versus TC+CC: OR = 1.14, 95% CI: 1.01–1.29, *P*_h_ = 0.69, *P* = 0.03) (Figure 2). However, no significant associations were found between the miR-149 polymorphism and colorectal cancer and gastric cancer in any of the genetic models when the analysis was stratified by cancer type. The results are shown in Table 3.

### Test of heterogeneity and sensitivity analysis

There was significant heterogeneity in the studies of miR-499. Then we evaluated the source of heterogeneity in in all comparisons and subgroups. After stratification, the heterogeneities decreased obviously in the subgroups of HCC, GC, CRC (*P*_h_ > 0.10 and I^2^ < 50% in most genetic comparisons) (Table 3). Further, meta-regression was used in our study. As shown in Table 4, all the factors extracted from the publications, including genotyping method (PCR-RFLP or HRMA), source of control (hospital based or population based), size (more than 500 hundred total number or else, *P* of HWE (*P* > 0.05 or else) were not the source of the heterogeneity .

**Table 4:** The Results of Meta-regression of rs3746444 (P).

Factors	GG vs AA	AG vs AA	G vs A	GG+AG vs AA	GG vs AG+AA
Source of control	0.11	0.10	0.08	0.07	0.13
Genotyping method	0.30	0.21	0.56	0.90	0.11
Size	0.32	0.56	0.60	0.93	0.29
HWE	0.58	0.37	0.85	0.83	0.44

**Figure 3:**
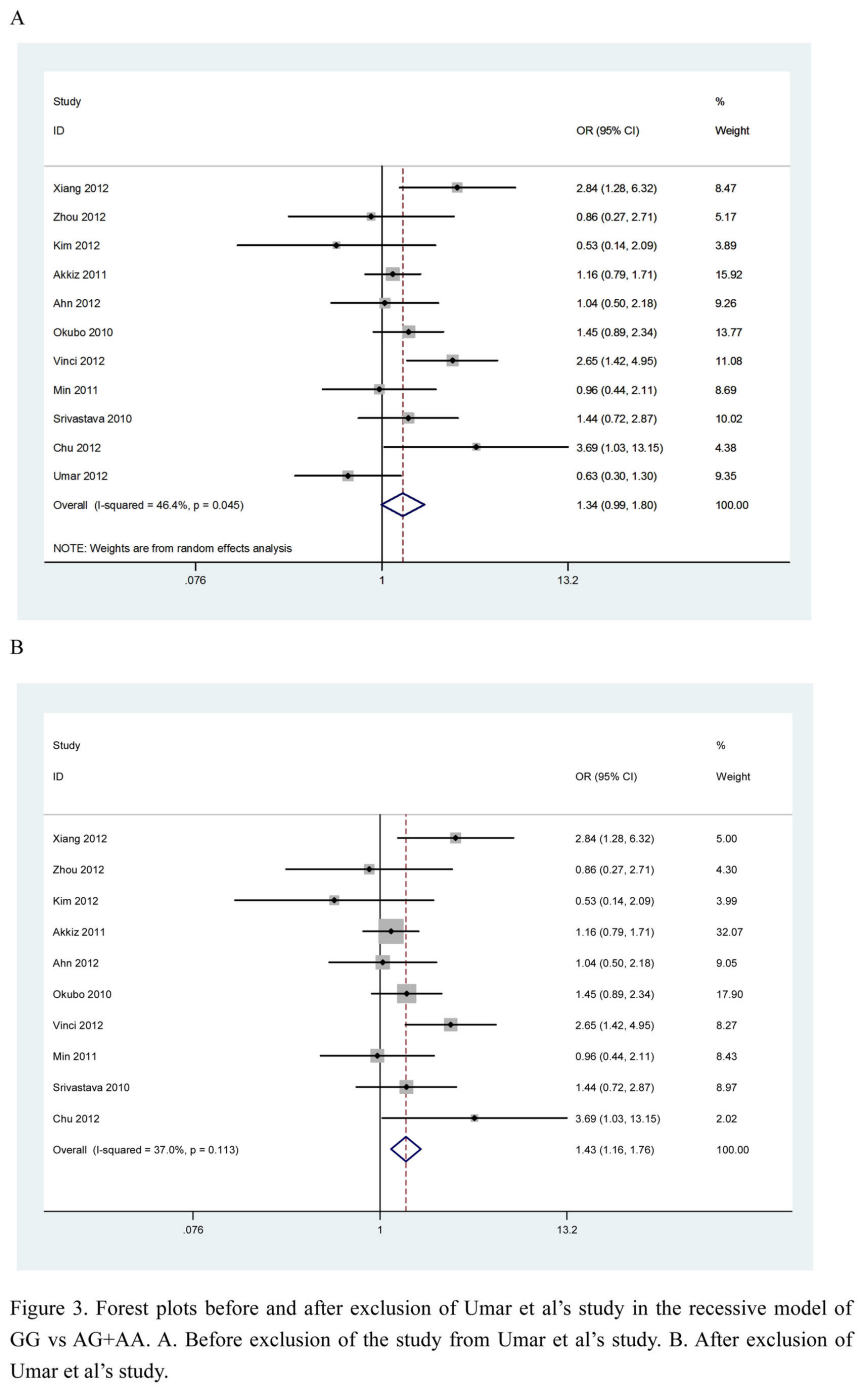
Forest plots before and after exclusion of Umar et al’s study in the recessive model of GG vs AG+AA. A. Before exclusion of the study from Umar et al’s study. B. After exclusion of Umar et al’s study.

Then sensitivity analysis was performed. We deleted one single study from the overall pooled analysis each time to check the influence of the removed data set to the overall ORs. For miR-499, one study [13] changed the between-study heterogeneity materially (AG vs. AA: Ph increased from 0.004 to 0.34, GG+AG vs. AA: Ph increased from 0.001 to 0.15). After exclusion of another study [18] in our study, the between-study heterogeneity and pooled ORs changed significantly. The results of homozygote comparison (GG vs AA) changed from OR = 1.33, 95% CI: 0.96–1.85, *P*_h_ = 0.003, *P* = 0.09 to OR = 1.45, 95% CI: 1.06–1.99, *P*_h_ = 0.08, *P* = 0.02. The results of recessive model (GG vs AG+AA) changed from OR = 1.34, 95% CI: 0.99–1.80, *P*_h_ = 0.04, *P* = 0.06 to OR = 1.43, 95% CI: 1.16–1.76, *P*_h_ = 0.11, *P* = 0.0006 (Figure 3). Caution should be made when interpreting the result of these two comparison. For miR-149, the between-study heterogeneity and pooled ORs were not materially altered.

### Publication bias

**Figure 4:**
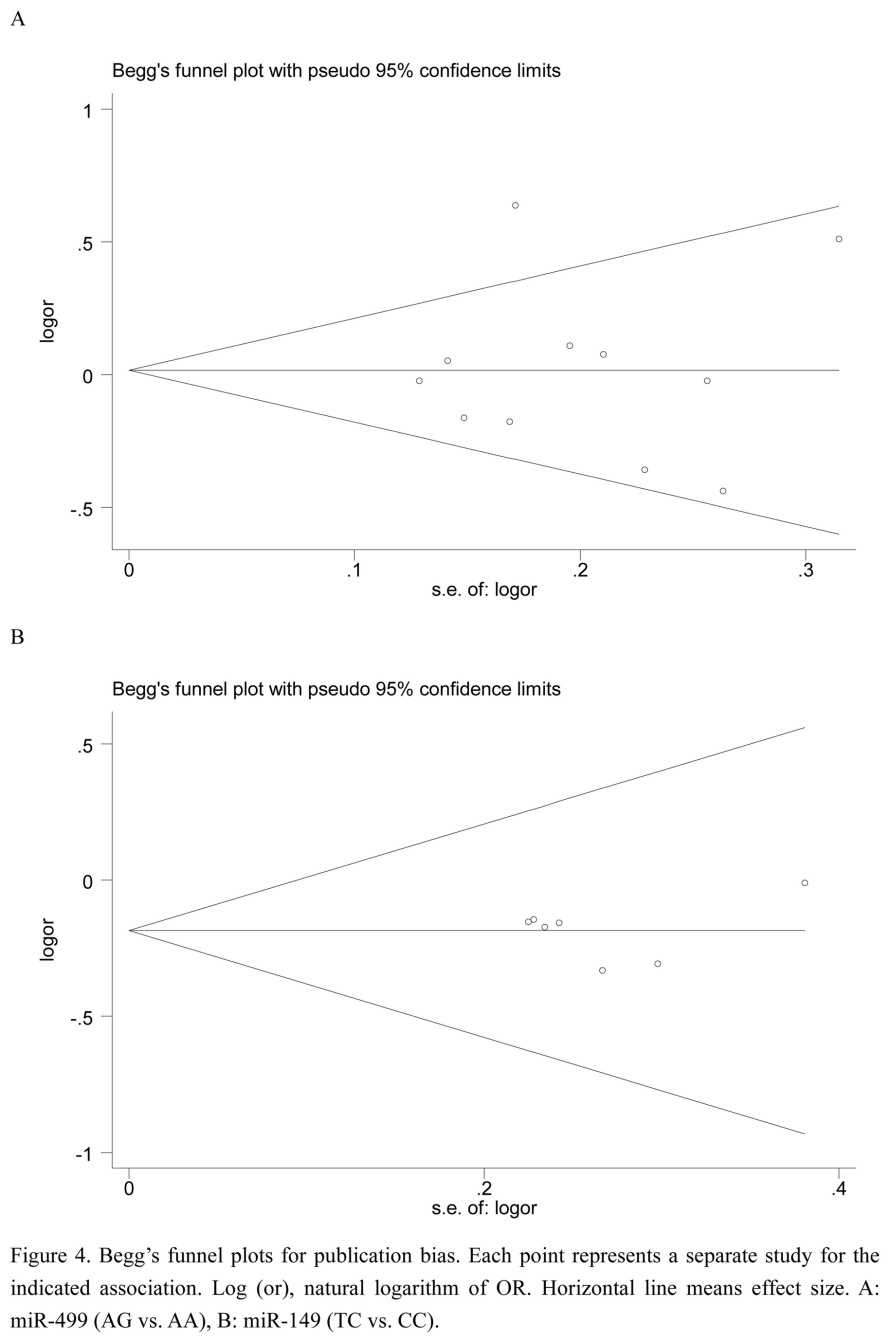
Begg’s funnel plots for publication bias. Each point represents a separate study for the indicated association. Log (or), natural logarithm of OR. Horizontal line means effect size. A: miR-499 (AG vs. AA), B: miR-149 (TC vs. CC).

We used Egger’s test and Begg’s test to access the publication bias of literatures in any comparison model for two SNPs. The result of Egger’s test did not show any statistically significant evidence for publication bias for the two SNPs (all *P*-values >  0.05, Table S3). And no obvious asymmetry was observed in Begg’s funnel plots (mir-499 rs3746444 , AG vs. AA, miR-149 (TC vs. CC) (Figure 4).

## Discussion

SNPs are the most common genetic sequence variation in human genome. These variations can affect the coding and splicing of miRNAs sequences, which can influence cancer susceptibility in a population [25]. SNPs in miRNAs can affect miRNA function by modulating the transcription of the primary transcript, pri-miRNA and pre-miRNA processing and maturation, or miRNA-miRNA interactions, potentially contributing to cancer susceptibility [26]. Some case-control studies and meta-analyses have revealed links between cancer risk and genetic variations in miRNA-coding regions. One meta-analysis reported that the miR-196a2 rs11614913 polymorphism may increase susceptibility to digestive system cancers [34]. The miR-146a rs2910164 and the miR-196a2 rs11614913 polymorphisms may not be associated with the risk of hepatocellular carcinoma [35]. The CC genotype of the has-miR-146a rs2910164 polymorphism was found to be associated with an increased risk of breast cancer in Europeans [36]. No significant associations between the miR-146a G/C polymorphism and cancer risk were identified when the eligible studies were pooled into the meta-analysis [37]. In this study, we performed a meta-analysis on all of the available published studies to examine the association between the miR-499 polymorphism (rs2910164) and the miR-149 polymorphism (rs292832) and susceptibility to gastrointestinal cancer in order to clarify conflicting results from previous reports. 

 MiR-499 deserves additional attention as an ideal biomarker for carcinogenesis due to its participation in biological processes such as cellular senescence, apoptosis, inflammation and the immune response, all of which are crucial in the development and progression of cancer [27-29]. A previous study of colorectal cancer found that the overexpression of miRNA- 499 may facilitate the migration and invasion of cancer cells in vitro, as well as metastasis to the lung and liver in vivo. Additionally, this study also identified forkhead box O4 (FOXO4) and programmed cell death 4 (PDCD4) as direct functional targets of miRNA499 [30]. Due to Umar er al.(ESCC) has an influence in pooling ORs for homozygote comparison and recessive model, the results of meta-analysis may have been impacted. The gene-environment interaction(smokers, tobacco chewers, drinkers, occupational exposure) may be the main factor. More well-designed studies based on larger sample sizes are needed to clear the association. In the subgroup analysis by cancer type, Umar er al.(ESCC) has no influence in CRC, HCC and GC. So the results were stabilized. Our meta-analysis showed a increased risk of colorectal cancer in individuals who are homozygous for the miR-499 polymorphism. This was the first meta-analysis to find that miR-499 contributes to susceptibility to colorectal cancer. However, we did not find any correlation between the miR-499 polymorphism and other types of cancer, including hepatocellular carcinoma and gastric cancer. 

 MiR-149 has been shown to function as both a tumor suppressor [31] and an oncogene [32] in the development of multiple types of solid tumors. MiR-149 may function as a tumor suppressor in gastric cancer cells and play an important role in inhibiting *ZBTB2*. Therefore, the downregulation of miR-149 promotes gastric cancer cell proliferation and cell cycle progression [33]. However, another study found that neither homozygotes nor heterozygotes with mutated miR-149 genotypes showed an increased risk of colorectal and gastric cancer [23]. The present meta-analysis explored the association between the miR-149 C>T polymorphism and overall cancer risk in the recessive model (TT versus TC+CC: OR = 1.15, 95% CI: 1.03–1.30, *P*_h_ = 0.68, *P* = 0.02). In addition, a marginally increased risk was found in Asian populations in the recessive models (TT versus TC+CC: OR = 1.14, 95% CI: 1.01–1.29, *P*_h_ = 0.79, *P* = 0.03). These two points were different from the previous meta-analysis. However, we failed to find an association between the miR-149 C>T polymorphism and gastric and colorectal cancers, among other cancer types. Because of only one study investigated Caucasians, we were unable to pool ORs. 

 This meta-analysis (3,275 cases and 3,794 controls for miR-499, 2,413 cases and 2,457 controls for miR-149)which can provide suitable statistical power and strengthen the reliability of our results. However, some limitations should be addressed. Firstly, only published studies were included in this meta-analysis, unpublished data and ongoing studies were not sought, which may have biased our results. Secondly, a lack of sufficient eligible studies limited our stratified analysis of additional types of cancer, such as esophageal squamous cell carcinoma, gallbladder cancer and oral cavity squamous cancer. Thirdly, potential gene-gene interaction and gene-environment interaction were evaluated in this meta-analysis, as no sufficient data could be extracted from the included studies. Fourthly, as with most meta-analyses, results should be interpreted with caution because of obvious between-study heterogeneity in some comparisons. Conﬁrming the role of the miR-499 rs3746444 polymorphism and miR-149 in GI cancer risk requires additional large studies in different populations and in different types of cancer.

 In conclusion, despite limited randomized controlled trials data available for particular comparisons, the miR-499 polymorphism (rs2910164) may be connected to increased susceptibility to colorectal cancer. The miR-149 polymorphism (rs292832) may marginally contribute to gastrointestinal cancer susceptibility, based on the pooled studies, especially for Asians. However, larger well-designed studies with subjects of the same ethnic background and biological characterization are warranted to validate these results.

## Supporting Information

Checklist S1 **PRISMA Checklist.**
(DOC)

Figure S1 **The data input into Cochrane Review Manager in machine readable form.**
(RM5)

Table S1 **Genotypes distribution of studies included for rs3746444 and rs2292832.**
(DOC)

Table S2 **Hardy-Weinberg Equilibrium Test for rs3746444 and rs2292832 in control groups.**
(DOC)

Table S3 **Egger’s test and Begg’s test of publication bias.**
(DOC)

Table S4 **The list of 69 studies considered and reasons for inclusion or exclusion.**
(DOC)
