# Supplementary material for: The Association between Two MicroRNA Variants (miR-499, miR-149) and Gastrointestinal Cancer Risk: A Meta-Analysis
Source: PLoS One. 2013 Nov 29;8(11):e81967. doi: 10.1371/journal.pone.0081967 (PMC3843688; doi:10.1371/journal.pone.0081967)
Supplement: Table S1 — Genotypes distribution of studies included for rs3746444 and rs2292832. (DOC) [file pone.0081967.s003.doc]

A

| **Author** | **Year** | **N(Cases/**  **Controls)** | **Cases** | | | | | **Controls** | | | | |
| --- | --- | --- | --- | --- | --- | --- | --- | --- | --- | --- | --- | --- |
|  |  |  | **AA(n)** | **AG(n)** | **GG(n)** | **A(n)** | **G(n)** | **AA(n)** | **AG(n)** | **GG(n)** | **A(n)** | **G(n)** |
| Xiang | 2012 | 100/100 | 36 | 40 | 24 | 112 | 88 | 54 | 36 | 10 | 144 | 56 |
| Zhou | 2012 | 186/483 | 141 | 41 | 4 | 323 | 49 | 371 | 100 | 12 | 842 | 124 |
| Kim | 2012 | 159/201 | 109 | 47 | 3 | 265 | 53 | 120 | 74 | 7 | 314 | 88 |
| Akkiz | 2011 | 222/222 | 45 | 87 | 90 | 177 | 267 | 47 | 93 | 82 | 187 | 257 |
| Ahn | 2012 | 461/447 | 323 | 123 | 15 | 769 | 153 | 299 | 134 | 14 | 732 | 162 |
| Okubo | 2010 | 552/697 | 364 | 151 | 37 | 879 | 225 | 466 | 198 | 33 | 1130 | 264 |
| Vinci | 2012 | 160/178 | 93 | 32 | 35 | 218 | 102 | 105 | 56 | 17 | 266 | 90 |
| Min | 2011 | 446/502 | 292 | 142 | 12 | 726 | 166 | 334 | 154 | 14 | 822 | 182 |
| Srivastava | 2010 | 230/230 | 112 | 97 | 21 | 321 | 139 | 121 | 94 | 15 | 366 | 124 |
| Chu | 2012 | 470/425 | 339 | 119 | 12 | 797 | 143 | 356 | 66 | 3 | 778 | 72 |
| Umar | 2012 | 289/309 | 155 | 122 | 12 | 432 | 146 | 149 | 140 | 20 | 438 | 180 |

| **Author** | **Year** | **N(Cases/**  **Controls)** | **Cases** | | | | | **Controls** | | | | |
| --- | --- | --- | --- | --- | --- | --- | --- | --- | --- | --- | --- | --- |
|  |  |  | **TT(n)** | **CC(n)** | **TC(n)** | **T(n)** | **C(n)** | **TT(n)** | **CC(n)** | **TC(n)** | **T(n)** | **C(n)** |
| Kim | 2012 | 159/201 | 81 | 14 | 64 | 226 | 92 | 83 | 21 | 97 | 263 | 139 |
| Ahn | 2012 | 461/447 | 241 | 44 | 176 | 658 | 264 | 220 | 40 | 187 | 627 | 267 |
| Min | 2011 | 446/502 | 221 | 48 | 177 | 619 | 273 | 232 | 51 | 219 | 683 | 321 |
| Chu | 2012 | 470/425 | 345 | 37 | 88 | 779 | 162 | 315 | 26 | 84 | 714 | 136 |
| Vinci | 2012 | 160/178 | 23 | 79 | 58 | 104 | 216 | 17 | 86 | 75 | 109 | 247 |
| Zhang* | 2012 | 435/443 | 203 | 50 | 190 | 596 | 290 | 187 | 46 | 202 | 576 | 294 |
| Zhang^&^ | 2012 | 274/269 | 132 | 41 | 101 | 365 | 183 | 114 | 35 | 120 | 348 | 190 |

B

Table S1 Genotypes distribution of studies included. A: rs3746444,

B: rs2292832.

*: Genotypes distribution of CRC.

^&^: Genotypes distribution of GC.
