# Supplementary material for: The Association between Two MicroRNA Variants (miR-499, miR-149) and Gastrointestinal Cancer Risk: A Meta-Analysis
Source: PLoS One. 2013 Nov 29;8(11):e81967. doi: 10.1371/journal.pone.0081967 (PMC3843688; doi:10.1371/journal.pone.0081967)
Supplement: Table S2 — Hardy-Weinberg Equilibrium Test for rs3746444 and rs2292832 in control groups. (DOC) [file pone.0081967.s004.doc]

Table S3. Egger’s test and Begg’s test of publication bias.

A

|  | Egger's test | | | | Begg's test |
| --- | --- | --- | --- | --- | --- |
|  | Coef | 95% CI | t | p | p |
| GG vs AA | -0.14 | (-3.57,3.30) | -0.09 | 0.93 | 0.64 |
| AG vs AA | 0.01 | (-4.38,4.41) | 0.01 | 0.99 | 1.00 |
| G vs A | 2.83 | (-3.50,9.15) | 1.01 | 0.34 | 0.35 |
| GG+AG vs AA | 1.32 | (-3.62,6.25) | 0.60 | 0.56 | 0.53 |
| GG vs AG+AA | -0.04 | (-2.80,2.72) | -0.03 | 0.98 | 0.64 |

B

|  | Egger's test | | | | Begg's test |
| --- | --- | --- | --- | --- | --- |
|  | Coef | 95% CI | t | p | p |
| TT vs CC | 2.50 | (-0.61,5.61) | 2.07 | 0.09 | 1.00 |
| TC vs CC | 0.10 | (-2.28,2.47) | 0.11 | 0.92 | 0.76 |
| T vs C | 1.31 | (-2.34,4.96) | 0.92 | 0.40 | 0.37 |
| TT+TC vs CC | 0.80 | (-2.34,3.94) | 0.66 | 0.54 | 0.76 |
| TT vs TC+CC | 2.09 | (-0.24,4.43) | 2.30 | 0.07 | 0.37 |

A: Egger’s test and Begg’s test for miR-499.

B: Egger’s test and Begg’s test for miR-149.
