# Supplementary material for: The Association between Two MicroRNA Variants (miR-499, miR-149) and Gastrointestinal Cancer Risk: A Meta-Analysis
Source: PLoS One. 2013 Nov 29;8(11):e81967. doi: 10.1371/journal.pone.0081967 (PMC3843688; doi:10.1371/journal.pone.0081967)
Supplement: Table S4 — The list of 69 studies considered and reasons for inclusion or exclusion. (DOC) [file pone.0081967.s006.doc]

69 studies were found from Jan. 1, 2000 to Jan. 1, 2013.

In the 69 studies, 6 studies were meta-analysis.

1. Srivastava K, Srivastava A (2012) Comprehensive review of genetic association studies and meta-Analyses on miRNA Polymorphisms and cancer risk. PLoS ONE 7(11): e50966. doi:10.1371/journal.pone.\0050966.
   2. He B, Pan Y, Cho WC, et al. (2012) The association between four genetic variants in microRNAs (rs11614913, rs2910164, rs3746444, rs2292832) and cancer risk: evidence from published studies. PLoS One. 7(11) pe49032
   3. Wang F, Sun G, Zou Y, et al.(2012) Association of microRNA-499 rs3746444 polymorphism with cancer risk: evidence from 7188 cases and 8548 controls. PLoS One. 7(9) pe45042.
   4. Qiu MT, Hu JW, Ding XX, et al.(2012) Hsa-miR-499 rs3746444 Polymorphism Contributes to Cancer Risk: A Meta-Analysis of 12 Studies [In Process Citation].PLoS One. 7(12) pe50887
   5. Zhang J, Liu YF, Gan Y (2012) Lack of association between miR-149 C>T polymorphism and cancer susceptibility: a meta-analysis based on 4,677 cases and 4,830 controls. Mol Biol Rep. Sep;39(9):8749-53. doi: 10.1007/s11033-012-1735-4. Epub 2012 Jun 20.
2. Zou P, Zhao L, Xu H, et al.(2012) [Hsa-mir-499 rs3746444 polymorphism and cancer risk: a meta-analysis.](http://www.ncbi.nlm.nih.gov/pubmed/23554757)J Biomed Res. 2012 Jul;26(4):253-9. doi: 10.7555/JBR.26.20110122. Epub 2012 Apr 16.

5 studies were review.

1. [Lafferty-Whyte K](http://www.ncbi.nlm.nih.gov/pubmed?term=Lafferty-Whyte K[Author]&cauthor=true&cauthor_uid=19419692), [Cairney CJ](http://www.ncbi.nlm.nih.gov/pubmed?term=Cairney CJ[Author]&cauthor=true&cauthor_uid=19419692), [Jamieson NB](http://www.ncbi.nlm.nih.gov/pubmed?term=Jamieson NB[Author]&cauthor=true&cauthor_uid=19419692), et al. (2009) Pathway analysis of senescence-associated miRNA targets reveals common processes to different senescence induction mechanisms. [Biochim Biophys Acta.](http://www.ncbi.nlm.nih.gov/pubmed/19419692)Apr;1792(4):341-52. doi: 10.1016/j.bbadis.2009.02.003. Epub 2009 Feb 12.

2. [Slaby O](http://www.ncbi.nlm.nih.gov/pubmed?term=Slaby O[Author]&cauthor=true&cauthor_uid=21692980), [Bienertova-Vasku J](http://www.ncbi.nlm.nih.gov/pubmed?term=Bienertova-Vasku J[Author]&cauthor=true&cauthor_uid=21692980), [Svoboda M](http://www.ncbi.nlm.nih.gov/pubmed?term=Svoboda M[Author]&cauthor=true&cauthor_uid=21692980), et al.(2012) Genetic polymorphisms and microRNAs: new direction in molecular epidemiology of solid cancer. [J Cell Mol Med.](http://www.ncbi.nlm.nih.gov/pubmed/?term=Genetic+polymorphisms+and+microRNAs:+new+direction+in+molecular+epidemiology+of+solid+cancer) Jan;16(1):8-21. doi: 10.1111/j.1582-4934.2011.01359.x.

3. [Fang Y](http://www.ncbi.nlm.nih.gov/pubmed?term=Fang Y[Author]&cauthor=true&cauthor_uid=22367141), [Fang D](http://www.ncbi.nlm.nih.gov/pubmed?term=Fang D[Author]&cauthor=true&cauthor_uid=22367141), [Hu J](http://www.ncbi.nlm.nih.gov/pubmed?term=Hu J[Author]&cauthor=true&cauthor_uid=22367141). et al. (2012) MicroRNA and its roles in esophageal cancer. [Med Sci Monit.](http://www.ncbi.nlm.nih.gov/pubmed/22367141) 2012 Mar;18(3):RA22-30.

4. [Song JH](http://www.ncbi.nlm.nih.gov/pubmed?term=Song JH[Author]&cauthor=true&cauthor_uid=22580099), [Meltzer SJ](http://www.ncbi.nlm.nih.gov/pubmed?term=Meltzer SJ[Author]&cauthor=true&cauthor_uid=22580099). et al. (2012) MicroRNAs in pathogenesis, diagnosis, and treatment of gastroesophageal cancers. [Gastroenterology.](http://www.ncbi.nlm.nih.gov/pubmed/22580099) Jul;143(1):35-47.e2. doi: 10.1053/j.gastro.2012.05.003. Epub 2012 May 10.

5. [Zhao X](http://www.ncbi.nlm.nih.gov/pubmed?term=Zhao X[Author]&cauthor=true&cauthor_uid=23108868), [Yang Z](http://www.ncbi.nlm.nih.gov/pubmed?term=Yang Z[Author]&cauthor=true&cauthor_uid=23108868), [Li G](http://www.ncbi.nlm.nih.gov/pubmed?term=Li G[Author]&cauthor=true&cauthor_uid=23108868),et al. (2012) The role and clinical implications of microRNAs in hepatocellular carcinoma.[Sci China Life Sci.](http://www.ncbi.nlm.nih.gov/pubmed/23108868) Oct;55(10):906-19. doi: 10.1007/s11427-012-4384-x. Epub 2012 Oct 31.

24 studies were not about GI cancers.

1. [Zhang M](http://www.ncbi.nlm.nih.gov/pubmed?term=Zhang M[Author]&cauthor=true&cauthor_uid=22074121), [Jin M](http://www.ncbi.nlm.nih.gov/pubmed?term=Jin M[Author]&cauthor=true&cauthor_uid=22074121), [Yu Y](http://www.ncbi.nlm.nih.gov/pubmed?term=Yu Y[Author]&cauthor=true&cauthor_uid=22074121), et al. (2012) Associations of miRNA polymorphisms and female physiological characteristics with breast cancer risk in Chinese population.[Eur J Cancer Care (Engl).](http://www.ncbi.nlm.nih.gov/pubmed/22074121) Mar;21(2):274-80. doi: 10.1111/j.1365-2354.2011.01308.x. Epub 2011 Nov 11.
2. .[Jin L](http://www.ncbi.nlm.nih.gov/pubmed?term=Jin L[Author]&cauthor=true&cauthor_uid=21896753), [Hu WL](http://www.ncbi.nlm.nih.gov/pubmed?term=Hu WL[Author]&cauthor=true&cauthor_uid=21896753), [Jiang CC](http://www.ncbi.nlm.nih.gov/pubmed?term=Jiang CC[Author]&cauthor=true&cauthor_uid=21896753), et al. (2011) MicroRNA-149, a p53-responsive microRNA, functions as an oncogenic regulator in human melanoma.[Proc Natl Acad Sci U S A.](http://www.ncbi.nlm.nih.gov/pubmed/21896753) Sep 20;108(38):15840-5. doi: 10.1073/pnas.1019312108. Epub 2011 Sep 6.
3. [Luo Z](http://www.ncbi.nlm.nih.gov/pubmed?term=Luo Z[Author]&cauthor=true&cauthor_uid=21873783), [Zhang L](http://www.ncbi.nlm.nih.gov/pubmed?term=Zhang L[Author]&cauthor=true&cauthor_uid=21873783), [Li Z](http://www.ncbi.nlm.nih.gov/pubmed?term=Li Z[Author]&cauthor=true&cauthor_uid=21873783),et al (2011) MiR-149 promotes epithelial-mesenchymal transition and invasion in nasopharyngeal carcinoma cells.[Zhong Nan Da Xue Xue Bao Yi Xue Ban.](http://www.ncbi.nlm.nih.gov/pubmed/21873783) Jul;36(7):604-9. doi: 10.3969/j.issn.1672-7347.2011.07.004.
4. [Liu H](http://www.ncbi.nlm.nih.gov/pubmed?term=Liu H[Author]&cauthor=true&cauthor_uid=20420713), [Brannon AR](http://www.ncbi.nlm.nih.gov/pubmed?term=Brannon AR[Author]&cauthor=true&cauthor_uid=20420713), [Reddy AR](http://www.ncbi.nlm.nih.gov/pubmed?term=Reddy AR[Author]&cauthor=true&cauthor_uid=20420713), et al. (2010) Identifying mRNA targets of microRNA dysregulated in cancer: with application to clear cell Renal Cell Carcinoma.[BMC Syst Biol.](http://www.ncbi.nlm.nih.gov/pubmed/20420713)Apr 27;4:51. doi: 10.1186/1752-0509-4-51.
5. [Schaefer A](http://www.ncbi.nlm.nih.gov/pubmed?term=Schaefer A[Author]&cauthor=true&cauthor_uid=19676045), [Jung M](http://www.ncbi.nlm.nih.gov/pubmed?term=Jung M[Author]&cauthor=true&cauthor_uid=19676045), [Mollenkopf HJ](http://www.ncbi.nlm.nih.gov/pubmed?term=Mollenkopf HJ[Author]&cauthor=true&cauthor_uid=19676045), et al.(2010) Diagnostic and prognostic implications of microRNA profiling in prostate carcinoma.[Int J Cancer.](http://www.ncbi.nlm.nih.gov/pubmed/19676045) Mar 1;126(5):1166-76. doi: 10.1002/ijc.24827.
6. [Hu Z](http://www.ncbi.nlm.nih.gov/pubmed?term=Hu Z[Author]&cauthor=true&cauthor_uid=18634034), [Liang J](http://www.ncbi.nlm.nih.gov/pubmed?term=Liang J[Author]&cauthor=true&cauthor_uid=18634034), [Wang Z](http://www.ncbi.nlm.nih.gov/pubmed?term=Wang Z[Author]&cauthor=true&cauthor_uid=18634034),er al.(2009) Common genetic variants in pre-microRNAs were associated with increased risk of breast cancer in Chinese women.[Hum Mutat.](http://www.ncbi.nlm.nih.gov/pubmed/18634034) Jan;30(1):79-84. doi: 10.1002/humu.20837.
7. [Zhang MW](http://www.ncbi.nlm.nih.gov/pubmed?term=Zhang MW[Author]&cauthor=true&cauthor_uid=21671485), [Yu YX](http://www.ncbi.nlm.nih.gov/pubmed?term=Yu YX[Author]&cauthor=true&cauthor_uid=21671485), [Jin MJ](http://www.ncbi.nlm.nih.gov/pubmed?term=Jin MJ[Author]&cauthor=true&cauthor_uid=21671485), et al. (2011) Association of miR-605 and miR-149 genetic polymorphisms with related risk factors of lung cancer susceptibility. [Zhejiang Da Xue Xue Bao Yi Xue Ban.](http://www.ncbi.nlm.nih.gov/pubmed/?term=Association+of+miR-605+and+miR-149+genetic+polymorphisms+with+related+risk+factors+of+lung+cancersusceptibility) May;40(3):265-71.[Article in Chinese]
8. [Rahman S](http://www.ncbi.nlm.nih.gov/pubmed?term=Rahman S[Author]&cauthor=true&cauthor_uid=22496815), [Quann K](http://www.ncbi.nlm.nih.gov/pubmed?term=Quann K[Author]&cauthor=true&cauthor_uid=22496815), [Pandya D](http://www.ncbi.nlm.nih.gov/pubmed?term=Pandya D[Author]&cauthor=true&cauthor_uid=22496815), et al. (2012) HTLV-1 Tax mediated downregulation of miRNAs associated with chromatin remodeling factors in T cells with stably integrated viral promoter.[PLoS One.](http://www.ncbi.nlm.nih.gov/pubmed/22496815) 2012;7(4):e34490. doi: 10.1371/journal.pone.0034490. Epub 2012 Apr 4.
9. [Vinci S](http://www.ncbi.nlm.nih.gov/pubmed?term=Vinci S[Author]&cauthor=true&cauthor_uid=21902575), [Gelmini S](http://www.ncbi.nlm.nih.gov/pubmed?term=Gelmini S[Author]&cauthor=true&cauthor_uid=21902575), [Pratesi N](http://www.ncbi.nlm.nih.gov/pubmed?term=Pratesi N[Author]&cauthor=true&cauthor_uid=21902575), et al.(2011)Genetic variants in miR-146a, miR-149, miR-196a2, miR-499 and their influence on relative expression in lung cancers.[Clin Chem Lab Med.](http://www.ncbi.nlm.nih.gov/pubmed/21902575) Sep 9;49(12):2073-80. doi: 10.1515/CCLM.2011.708.
10. [Mittal RD](http://www.ncbi.nlm.nih.gov/pubmed?term=Mittal RD[Author]&cauthor=true&cauthor_uid=21345130), [Gangwar R](http://www.ncbi.nlm.nih.gov/pubmed?term=Gangwar R[Author]&cauthor=true&cauthor_uid=21345130), [George GP](http://www.ncbi.nlm.nih.gov/pubmed?term=George GP[Author]&cauthor=true&cauthor_uid=21345130),et al (2011)Investigative role of pre-microRNAs in bladder cancer patients: a case-control study in North India.[DNA Cell Biol.](http://www.ncbi.nlm.nih.gov/pubmed/21345130)Jun;30(6):401-6. doi: 10.1089/dna.2010.1159. Epub 2011 Feb 23.
11. [Zhou B](http://www.ncbi.nlm.nih.gov/pubmed?term=Zhou B[Author]&cauthor=true&cauthor_uid=21319225), [Wang K](http://www.ncbi.nlm.nih.gov/pubmed?term=Wang K[Author]&cauthor=true&cauthor_uid=21319225), [Wang Y](http://www.ncbi.nlm.nih.gov/pubmed?term=Wang Y[Author]&cauthor=true&cauthor_uid=21319225), et al. (2011) Common genetic polymorphisms in pre-microRNAs and risk of cervical squamous cell carcinoma.[Mol Carcinog.](http://www.ncbi.nlm.nih.gov/pubmed/21319225) Jul;50(7):499-505. doi: 10.1002/mc.20740. Epub 2011 Feb 11.
12. [Liu Z](http://www.ncbi.nlm.nih.gov/pubmed?term=Liu Z[Author]&cauthor=true&cauthor_uid=20549817), [Li G](http://www.ncbi.nlm.nih.gov/pubmed?term=Li G[Author]&cauthor=true&cauthor_uid=20549817), [Wei S](http://www.ncbi.nlm.nih.gov/pubmed?term=Wei S[Author]&cauthor=true&cauthor_uid=20549817), et al. (2010) Genetic variants in selected pre-microRNA genes and the risk of squamous cell carcinoma of the head and neck.[Cancer.](http://www.ncbi.nlm.nih.gov/pubmed/20549817) Oct 15;116(20):4753-60. doi: 10.1002/cncr.25323.
13. [Catucci I](http://www.ncbi.nlm.nih.gov/pubmed?term=Catucci I[Author]&cauthor=true&cauthor_uid=19847796), [Yang R](http://www.ncbi.nlm.nih.gov/pubmed?term=Yang R[Author]&cauthor=true&cauthor_uid=19847796), [Verderio P](http://www.ncbi.nlm.nih.gov/pubmed?term=Verderio P[Author]&cauthor=true&cauthor_uid=19847796), et al. (2010) Evaluation of SNPs in miR-146a, miR196a2 and miR-499 as low-penetrance alleles in German and Italian familial breast cancer cases.[Hum Mutat.](http://www.ncbi.nlm.nih.gov/pubmed/19847796) Jan;31(1):E1052-7. doi: 10.1002/humu.21141.
14. [Hu Z](http://www.ncbi.nlm.nih.gov/pubmed?term=Hu Z[Author]&cauthor=true&cauthor_uid=18634034), [Liang J](http://www.ncbi.nlm.nih.gov/pubmed?term=Liang J[Author]&cauthor=true&cauthor_uid=18634034), [Wang Z](http://www.ncbi.nlm.nih.gov/pubmed?term=Wang Z[Author]&cauthor=true&cauthor_uid=18634034),et al. (2009) Common genetic variants in pre-microRNAs were associated with increased risk of breast cancer in Chinese women.[Hum Mutat.](http://www.ncbi.nlm.nih.gov/pubmed/18634034) Jan;30(1):79-84. doi: 10.1002/humu.20837.
15. [Pan SJ](http://www.ncbi.nlm.nih.gov/pubmed?term=Pan SJ[Author]&cauthor=true&cauthor_uid=23298478), [Zhan SK](http://www.ncbi.nlm.nih.gov/pubmed?term=Zhan SK[Author]&cauthor=true&cauthor_uid=23298478), [Pei BG](http://www.ncbi.nlm.nih.gov/pubmed?term=Pei BG[Author]&cauthor=true&cauthor_uid=23298478),et al. (2012) MicroRNA-149 inhibits proliferation and invasion of glioma cells via blockade of AKT1 signaling.[Int J Immunopathol Pharmacol.](http://www.ncbi.nlm.nih.gov/pubmed/23298478) Oct-Dec;25(4):871-81.
16. [Tu HF](http://www.ncbi.nlm.nih.gov/pubmed?term=Tu HF[Author]&cauthor=true&cauthor_uid=23272122), [Liu CJ](http://www.ncbi.nlm.nih.gov/pubmed?term=Liu CJ[Author]&cauthor=true&cauthor_uid=23272122), [Chang CL](http://www.ncbi.nlm.nih.gov/pubmed?term=Chang CL[Author]&cauthor=true&cauthor_uid=23272122),The association between genetic polymorphism and the processing efficiency of miR-149 affects the prognosis of patients with head and neck squamous cell carcinoma.[PLoS One.](http://www.ncbi.nlm.nih.gov/pubmed/23272122)7(12):e51606. doi: 10.1371/journal.pone.0051606. Epub 2012 Dec 14.
17. [Li D](http://www.ncbi.nlm.nih.gov/pubmed?term=Li D[Author]&cauthor=true&cauthor_uid=21978395), [Chen P](http://www.ncbi.nlm.nih.gov/pubmed?term=Chen P[Author]&cauthor=true&cauthor_uid=21978395), [Li XY](http://www.ncbi.nlm.nih.gov/pubmed?term=Li XY[Author]&cauthor=true&cauthor_uid=21978395), et al. (2010) Grade-specific expression profiles of miRNAs/mRNAs and docking study in human grade I-III astrocytomas.[OMICS.](http://www.ncbi.nlm.nih.gov/pubmed/21978395) Oct;15(10):673-82. doi: 10.1089/omi.2011.0064.
18. [Chen P](http://www.ncbi.nlm.nih.gov/pubmed?term=Chen P[Author]&cauthor=true&cauthor_uid=23053947), [Zhang J](http://www.ncbi.nlm.nih.gov/pubmed?term=Zhang J[Author]&cauthor=true&cauthor_uid=23053947), [Zhou F](http://www.ncbi.nlm.nih.gov/pubmed?term=Zhou F[Author]&cauthor=true&cauthor_uid=23053947) (2012) MiR-499 rs3746444 polymorphism is associated with cancer development among Asians and related to breast cancer susceptibility.[Mol Biol Rep.](http://www.ncbi.nlm.nih.gov/pubmed/?term=miR-499+rs3746444+polymorphism+is+associated+with+cancer+development+among+Asians+and+related+to+breast+cancer+susceptibility++0+推荐) Dec;39(12):10433-8. doi: 10.1007/s11033-012-1922-3. Epub 2012 Oct 10.
19. [Sand M](http://www.ncbi.nlm.nih.gov/pubmed?term=Sand M[Author]&cauthor=true&cauthor_uid=23026055), [Skrygan M](http://www.ncbi.nlm.nih.gov/pubmed?term=Skrygan M[Author]&cauthor=true&cauthor_uid=23026055), [Georgas D](http://www.ncbi.nlm.nih.gov/pubmed?term=Georgas D[Author]&cauthor=true&cauthor_uid=23026055), et al. (2012) Microarray analysis of microRNA expression in cutaneous squamous cell carcinoma.[J Dermatol Sci.](http://www.ncbi.nlm.nih.gov/pubmed/23026055) Dec;68(3):119-26. doi: 10.1016/j.jdermsci.2012.09.004. Epub 2012 Sep 13.
20. [George GP](http://www.ncbi.nlm.nih.gov/pubmed?term=George GP[Author]&cauthor=true&cauthor_uid=20842445), [Gangwar R](http://www.ncbi.nlm.nih.gov/pubmed?term=Gangwar R[Author]&cauthor=true&cauthor_uid=20842445), [Mandal RK](http://www.ncbi.nlm.nih.gov/pubmed?term=Mandal RK[Author]&cauthor=true&cauthor_uid=20842445), et al. (2011) Genetic variation in microRNA genes and prostate cancer risk in North Indian population. [Mol Biol Rep.](http://www.ncbi.nlm.nih.gov/pubmed/20842445) Mar;38(3):1609-15. doi: 10.1007/s11033-010-0270-4. Epub 2010 Sep 15.
21. [Tian T](http://www.ncbi.nlm.nih.gov/pubmed?term=Tian T[Author]&cauthor=true&cauthor_uid=19293314), [Shu Y](http://www.ncbi.nlm.nih.gov/pubmed?term=Shu Y[Author]&cauthor=true&cauthor_uid=19293314), [Chen J](http://www.ncbi.nlm.nih.gov/pubmed?term=Chen J[Author]&cauthor=true&cauthor_uid=19293314), et al.( 2009) A functional genetic variant in microRNA-196a2 is associated with increased susceptibility of lung cancer in Chinese.[Cancer Epidemiol Biomarkers Prev.](http://www.ncbi.nlm.nih.gov/pubmed/19293314) Apr;18(4):1183-7. doi: 10.1158/1055-9965.EPI-08-0814. Epub 2009 Mar 17.
22. [Heneghan HM](http://www.ncbi.nlm.nih.gov/pubmed?term=Heneghan HM[Author]&cauthor=true&cauthor_uid=20134314), [Miller N](http://www.ncbi.nlm.nih.gov/pubmed?term=Miller N[Author]&cauthor=true&cauthor_uid=20134314), [Lowery AJ](http://www.ncbi.nlm.nih.gov/pubmed?term=Lowery AJ[Author]&cauthor=true&cauthor_uid=20134314), et al. (2010) Circulating microRNAs as novel minimally invasive biomarkers for breast cancer. [Ann Surg.](http://www.ncbi.nlm.nih.gov/pubmed/20134314) Mar;251(3):499-505. doi: 10.1097/SLA.0b013e3181cc939f.
23. [Alshatwi AA](http://www.ncbi.nlm.nih.gov/pubmed?term=Alshatwi AA[Author]&cauthor=true&cauthor_uid=22363415), [Shafi G](http://www.ncbi.nlm.nih.gov/pubmed?term=Shafi G[Author]&cauthor=true&cauthor_uid=22363415), [Hasan TN](http://www.ncbi.nlm.nih.gov/pubmed?term=Hasan TN[Author]&cauthor=true&cauthor_uid=22363415), et al. (2012) Differential expression profile and genetic variants of microRNAs sequences in breast cancer patients. [PLoS One.](http://www.ncbi.nlm.nih.gov/pubmed/22363415)7(2):e30049. doi: 10.1371/journal.pone.0030049. Epub 2012 Feb 20.
24. Ling XX, Li YY,Yang L,et al. (2011) Genetic variant in seed region of hsa- miR- 499- 3p( rs3746444 A ＞ G) increases risk of lung cancer. Chin J Public Health Sep. Vol． 27. No．09- 1105- 03. [Article in Chinese]

19 studies had no control groups or about [fundamental research](app:ds:fundamental research).

1. Wang Y, Zheng X,Zhang Z,et al. (2012) MicroRNA-149 inhibits proliferation and cell cycle progression through the targeting of ZBTB2 in human gastric cancer.Plos One. 7(10):e41693. doi: 10.1371/journal.pone.0041693. Epub 2012 Oct 29.
2. [Jaiswal R](http://www.ncbi.nlm.nih.gov/pubmed?term=Jaiswal R[Author]&cauthor=true&cauthor_uid=22682234), [Luk F](http://www.ncbi.nlm.nih.gov/pubmed?term=Luk F[Author]&cauthor=true&cauthor_uid=22682234), [Gong J](http://www.ncbi.nlm.nih.gov/pubmed?term=Gong J[Author]&cauthor=true&cauthor_uid=22682234), et al.(2012) Microparticle conferred microRNA profiles--implications in the transfer and dominance of cancer traits. [Mol Cancer.](http://www.ncbi.nlm.nih.gov/pubmed/22682234) Jun 8;11:37. doi: 10.1186/1476-4598-11-37.
3. [Wong TS](http://www.ncbi.nlm.nih.gov/pubmed?term=Wong TS[Author]&cauthor=true&cauthor_uid=18451220), [Liu XB](http://www.ncbi.nlm.nih.gov/pubmed?term=Liu XB[Author]&cauthor=true&cauthor_uid=18451220), [Wong BY](http://www.ncbi.nlm.nih.gov/pubmed?term=Wong BY[Author]&cauthor=true&cauthor_uid=18451220), et al. (2008) Mature miR-184 as Potential Oncogenic microRNA of Squamous Cell Carcinoma of Tongue.[Clin Cancer Res.](http://www.ncbi.nlm.nih.gov/pubmed/?term=mir-149+and+oral+cancer) May 1;14(9):2588-92. doi: 10.1158/1078-0432.CCR-07-0666.
4. [Liu X](http://www.ncbi.nlm.nih.gov/pubmed?term=Liu X[Author]&cauthor=true&cauthor_uid=20006370), [Wang T](http://www.ncbi.nlm.nih.gov/pubmed?term=Wang T[Author]&cauthor=true&cauthor_uid=20006370), [Wakita T](http://www.ncbi.nlm.nih.gov/pubmed?term=Wakita T[Author]&cauthor=true&cauthor_uid=20006370), et al. (2010) Systematic identification of microRNA and messenger RNA profiles in hepatitis C virus-infected human hepatoma cells.[Virology.](http://www.ncbi.nlm.nih.gov/pubmed/20006370) Mar 1;398(1):57-67. doi: 10.1016/j.virol.2009.11.036. Epub 2009 Dec 14.
5. [Luo Z](http://www.ncbi.nlm.nih.gov/pubmed?term=Luo Z[Author]&cauthor=true&cauthor_uid=22260379), [Zhang L](http://www.ncbi.nlm.nih.gov/pubmed?term=Zhang L[Author]&cauthor=true&cauthor_uid=22260379), [Li Z](http://www.ncbi.nlm.nih.gov/pubmed?term=Li Z[Author]&cauthor=true&cauthor_uid=22260379),et al (2012) An in silico analysis of dynamic changes in microRNA expression profiles in stepwise development of nasopharyngeal carcinoma. [BMC Med Genomics.](http://www.ncbi.nlm.nih.gov/pubmed/22260379) Jan 19;5:3. doi: 10.1186/1755-8794-5-3.
6. [Lin RJ](http://www.ncbi.nlm.nih.gov/pubmed?term=Lin RJ[Author]&cauthor=true&cauthor_uid=20623644), [Lin YC](http://www.ncbi.nlm.nih.gov/pubmed?term=Lin YC[Author]&cauthor=true&cauthor_uid=20623644), [Yu AL](http://www.ncbi.nlm.nih.gov/pubmed?term=Yu AL[Author]&cauthor=true&cauthor_uid=20623644). et al. (2010) MiR-149 induces apoptosis by inhibiting Akt1 and E2F1 in human cancer cells.[Mol Carcinog.](http://www.ncbi.nlm.nih.gov/pubmed/20623644) 2010 Aug;49(8):719-27. doi: 10.1002/mc.20647.
7. [Wei W](http://www.ncbi.nlm.nih.gov/pubmed?term=Wei W[Author]&cauthor=true&cauthor_uid=22664953), [Hu Z](http://www.ncbi.nlm.nih.gov/pubmed?term=Hu Z[Author]&cauthor=true&cauthor_uid=22664953), [Fu H](http://www.ncbi.nlm.nih.gov/pubmed?term=Fu H[Author]&cauthor=true&cauthor_uid=22664953), et al. (2012) MicroRNA-1 and microRNA-499 downregulate the expression of the ets1 proto-oncogene in HepG2 cells.[Oncol Rep.](http://www.ncbi.nlm.nih.gov/pubmed/22664953) Aug;28(2):701-6. doi: 10.3892/or.2012.1850. Epub 2012 Jun 1.
8. [Liu X](http://www.ncbi.nlm.nih.gov/pubmed?term=Liu X[Author]&cauthor=true&cauthor_uid=21934092), [Zhang Z](http://www.ncbi.nlm.nih.gov/pubmed?term=Zhang Z[Author]&cauthor=true&cauthor_uid=21934092), [Sun L](http://www.ncbi.nlm.nih.gov/pubmed?term=Sun L[Author]&cauthor=true&cauthor_uid=21934092),et al. (2011) MicroRNA-499-5p promotes cellular invasion and tumor metastasis in colorectal cancer by targeting FOXO4 and PDCD4.[Carcinogenesis.](http://www.ncbi.nlm.nih.gov/pubmed/?term=MicroRNA-499-5p+Promotes+Cellular+Invasion+and+Tumor+Metastasis+in+Colorectal+Cancer+by+Targeting+FOXO4+and+PDCD4)Dec;32(12):1798-805. doi: 10.1093/carcin/bgr213. Epub 2011 Sep 20.
9. [Yan Z](http://www.ncbi.nlm.nih.gov/pubmed?term=Yan Z[Author]&cauthor=true&cauthor_uid=23007704), [Xiong Y](http://www.ncbi.nlm.nih.gov/pubmed?term=Xiong Y[Author]&cauthor=true&cauthor_uid=23007704), [Xu W](http://www.ncbi.nlm.nih.gov/pubmed?term=Xu W[Author]&cauthor=true&cauthor_uid=23007704),et al. (2012) Identification of recurrence-related genes by integrating microRNA and gene expression profiling of gastric cancer.[Int J Oncol.](http://www.ncbi.nlm.nih.gov/pubmed/23007704) Dec;41(6):2166-74. doi: 10.3892/ijo.2012.1637. Epub 2012 Sep 24.
10. [Osawa S](http://www.ncbi.nlm.nih.gov/pubmed?term=Osawa S[Author]&cauthor=true&cauthor_uid=22848236), [Shimada Y](http://www.ncbi.nlm.nih.gov/pubmed?term=Shimada Y[Author]&cauthor=true&cauthor_uid=22848236), [Sekine S](http://www.ncbi.nlm.nih.gov/pubmed?term=Sekine S[Author]&cauthor=true&cauthor_uid=22848236), et al. (2011) MicroRNA profiling of gastric cancer patients from formalin-fixed paraffin-embedded samples.
11. [Zhang Y](http://www.ncbi.nlm.nih.gov/pubmed?term=Zhang Y[Author]&cauthor=true&cauthor_uid=22821565), [Fan KJ](http://www.ncbi.nlm.nih.gov/pubmed?term=Fan KJ[Author]&cauthor=true&cauthor_uid=22821565), [Sun Q](http://www.ncbi.nlm.nih.gov/pubmed?term=Sun Q[Author]&cauthor=true&cauthor_uid=22821565), et al. (2012) Functional screening for miRNAs targeting Smad4 identified miR-199a as a negative regulator of TGF-β signalling pathway.[Nucleic Acids Res.](http://www.ncbi.nlm.nih.gov/pubmed/22821565) Oct;40(18):9286-97. doi: 10.1093/nar/gks667. Epub 2012 Jul 19.
12. [Li SC](http://www.ncbi.nlm.nih.gov/pubmed?term=Li SC[Author]&cauthor=true&cauthor_uid=22369582), [Liao YL](http://www.ncbi.nlm.nih.gov/pubmed?term=Liao YL[Author]&cauthor=true&cauthor_uid=22369582), [Ho MR](http://www.ncbi.nlm.nih.gov/pubmed?term=Ho MR[Author]&cauthor=true&cauthor_uid=22369582),et al. (2012) MiRNA arm selection and isomiR distribution in gastric cancer.[BMC Genomics.](http://www.ncbi.nlm.nih.gov/pubmed/22369582) 13 Suppl 1:S13. doi: 10.1186/1471-2164-13-S1-S13. Epub 2012 Jan 17.
13. [Lu L](http://www.ncbi.nlm.nih.gov/pubmed?term=Lu L[Author]&cauthor=true&cauthor_uid=22145532), [Li Y](http://www.ncbi.nlm.nih.gov/pubmed?term=Li Y[Author]&cauthor=true&cauthor_uid=22145532), [Li S](http://www.ncbi.nlm.nih.gov/pubmed?term=Li S[Author]&cauthor=true&cauthor_uid=22145532).(2011) Computational identification of potential microRNA network biomarkers for the progression stages of gastric cancer.[Int J Data Min Bioinform.](http://www.ncbi.nlm.nih.gov/pubmed/22145532) 5(5):519-31.
14. [Golestaneh AF](http://www.ncbi.nlm.nih.gov/pubmed?term=Golestaneh AF[Author]&cauthor=true&cauthor_uid=22374783), [Atashi A](http://www.ncbi.nlm.nih.gov/pubmed?term=Atashi A[Author]&cauthor=true&cauthor_uid=22374783), [Langroudi L](http://www.ncbi.nlm.nih.gov/pubmed?term=Langroudi L[Author]&cauthor=true&cauthor_uid=22374783), et al.(2012) MiRNAs expressed differently in cancer stem cells and cancer cells of human gastric cancer cell line MKN-45.[Cell Biochem Funct.](http://www.ncbi.nlm.nih.gov/pubmed/22374783) Jul;30(5):411-8. doi: 10.1002/cbf.2815. Epub 2012 Feb 28.
15. [Wu XM](http://www.ncbi.nlm.nih.gov/pubmed?term=Wu XM[Author]&cauthor=true&cauthor_uid=21293479), [Shao XQ](http://www.ncbi.nlm.nih.gov/pubmed?term=Shao XQ[Author]&cauthor=true&cauthor_uid=21293479), [Meng XX](http://www.ncbi.nlm.nih.gov/pubmed?term=Meng XX[Author]&cauthor=true&cauthor_uid=21293479),et al .(2011) Genome-wide analysis of microRNA and mRNA expression signatures in hydroxycamptothecin-resistant gastric cancer cells.[Acta Pharmacol Sin.](http://www.ncbi.nlm.nih.gov/pubmed/21293479) Feb;32(2):259-69. doi: 10.1038/aps.2010.204.
16. [Noto JM](http://www.ncbi.nlm.nih.gov/pubmed?term=Noto JM[Author]&cauthor=true&cauthor_uid=22919587), [Peek RM](http://www.ncbi.nlm.nih.gov/pubmed?term=Peek RM[Author]&cauthor=true&cauthor_uid=22919587) .(2012)The role of microRNAs in Helicobacter pylori pathogenesis and gastric carcinogenesis.[Front Cell Infect Microbiol.](http://www.ncbi.nlm.nih.gov/pubmed/22919587)Jan 3;1:21. doi: 10.3389/fcimb.2011.00021. eCollection 2011.
17. [Wang J](http://www.ncbi.nlm.nih.gov/pubmed?term=Wang J[Author]&cauthor=true&cauthor_uid=20797817), [Wang Q](http://www.ncbi.nlm.nih.gov/pubmed?term=Wang Q[Author]&cauthor=true&cauthor_uid=20797817), [Liu H](http://www.ncbi.nlm.nih.gov/pubmed?term=Liu H[Author]&cauthor=true&cauthor_uid=20797817),et al. (2010) MicroRNA expression and its implication for the diagnosis and therapeutic strategies of gastric cancer.[Cancer Lett.](http://www.ncbi.nlm.nih.gov/pubmed/20797817) Nov 28;297(2):137-43. doi: 10.1016/j.canlet.2010.07.018. Epub 2010 Aug 24.
18. [Aslam MI](http://www.ncbi.nlm.nih.gov/pubmed?term=Aslam MI[Author]&cauthor=true&cauthor_uid=22716183), [Patel M](http://www.ncbi.nlm.nih.gov/pubmed?term=Patel M[Author]&cauthor=true&cauthor_uid=22716183), [Singh B](http://www.ncbi.nlm.nih.gov/pubmed?term=Singh B[Author]&cauthor=true&cauthor_uid=22716183),et al.(2012) MicroRNA manipulation in colorectal cancer cells: from laboratory to clinical application.[J Transl Med.](http://www.ncbi.nlm.nih.gov/pubmed/22716183) Jun 20;10:128. doi: 10.1186/1479-5876-10-128.
19. [Lin M](http://www.ncbi.nlm.nih.gov/pubmed?term=Lin M[Author]&cauthor=true&cauthor_uid=22661538), [Gu J](http://www.ncbi.nlm.nih.gov/pubmed?term=Gu J[Author]&cauthor=true&cauthor_uid=22661538), [Eng C](http://www.ncbi.nlm.nih.gov/pubmed?term=Eng C[Author]&cauthor=true&cauthor_uid=22661538), et al. (2012) Genetic polymorphisms in MicroRNA-related genes as predictors of clinical outcomes in colorectal adenocarcinoma patients.[Clin Cancer Res.](http://www.ncbi.nlm.nih.gov/pubmed/22661538) Jul 15;18(14):3982-91. doi: 10.1158/1078-0432.CCR-11-2951. Epub 2012 Jun 1.

12 eligible studies based on the rearch critercia.

1. Xiang Y, Fan S, Cao J, Huang S, Zhang LP (2012) Association of the microRNA-499 variants with susceptibility to hepatocellular carcinoma in a Chinese population. Mol Biol Rep 39:7019–7023.
2. Kim WH, Min KT, Jeon YJ,et al. (2012) Association study of microRNA polymorphisms with hepatocellular carcinoma in Korean population. Gene 504(1):92-97.
3. Chu YH, Tzeng SL, Lin CW, et al. (2012) Impacts of microRNA gene polymorphisms on the susceptibility of environmental factors leading to carcinogenesis in oral cancer. PLoS One 7(6): e39777.
4. Srivastava K, Srivastava A, Mittal B (2010) Common genetic variants in pre-microRNAs and risk of gallbladder cancer in North Indian population. J Hum Genet 55(8):495-499.
5. Akkız H, Bayram S, Bayram S, Üsküdar O (2011) Genetic variation in the microRNA-499

gene and hepatoc, ellular carcinoma risk in a Turkish population: lack of any association in a case-control study. Asian Pac. J Cancer Prev 12(11):3107-12.

1. Umar M, Upadhyay R, Prakash G, et al. (2012) Evaluation of common genetic variants in pre-microRNA in susceptibility and prognosis of esophageal cancer. Mol Carcinog doi: 10.1002/mc.21931. Epub ahead of print.
2. Okubo M, Tahara T, Shibata T, et al. (2010)Association between common genetic variants inPre-microRNAs and gastric cancer risk in Japanese population. Helicobacter 15(6): 524-531.

8. Zhou J, Lv R, Song X, et al. (2012) A ssociation between two genatic variants in miRNA and primary liver cancer risk in the Chinese population. DNA and Cell Biology 31(4): 524-530.

9. Ahn DH, Rah H, Choi YK, Jeon YJ, Min KT, et al. (2012) Association of the miR-146aC>G, miR-149T>C,miR-196a2T>C, and miR-499A>G polymorphisms with gastric cancer risk and survival in the Korean population. Mol Carcinog doi: 10.1002/mc.21962, Epub ahead of print.

10. Min KT, Kim JW, Jeon YJ, Jang MJ, Chong SY, et al. (2012) Association of the miR-146aC>G,149C>T, 196a2C>T,and 499A>G polymorphisms with colorectal cancer in the Korean population. Mol Carcinog 51 Suppl 1: E65-73.

11. Vinci S, Gelmini S, Mancini I, Malentacchi F, Pazzagli M, et al. (2012) Genetic and epigenetic factors in regulation of microRNA in colorectal cancers. Methods pii: S1046-2023(12)00242-3.

12. Zhang MW, Jin MJ, Yu YX, Zhang SC, Liu B, et al. (2011) Associations of lifestyle-related factors, hsa-miR-149 and hsa-miR-605 gene polymorphisms with gastrointestinal cancer risk. Mol Carcinog doi: 10.1002/mc.20863. Epub 2011 Oct 4.
